# A Novel Finding of Lichenoid Drug Reaction From Ibrutinib Therapy

**DOI:** 10.7759/cureus.22433

**Published:** 2022-02-21

**Authors:** Merin Reji, Kinchit K Shah

**Affiliations:** 1 Internal Medicine, Wake Forest Baptist Medical Center, Winston Salem, USA; 2 Internal Medicine, Wake Forest Baptist Medical Center, Winston-Salem, USA

**Keywords:** allergy, hematology, lichenoid, drug reaction, ibrutinib

## Abstract

Ibrutinib is Burton’s tyrosine kinase inhibitor used in the treatment of chronic lymphocytic leukemia and other conditions. This drug has been generally thought to be well tolerated, although studies have identified a few distinct adverse effects, including gastrointestinal discomfort, nausea, diarrhea, and atrial fibrillation. Regarding cutaneous findings, ibrutinib has only been linked to two self-limiting forms of mild rashes generally. In this report, we discuss a lichenoid drug reaction to ibrutinib in a patient with chronic lymphocytic leukemia severe enough to result in drug discontinuation. This adverse effect has not been previously reported to our knowledge.

## Introduction

The development of target drug therapy, such as ibrutinib, has been a turning point in cancer treatment. Chronic lymphocytic leukemia (CLL) is a disease that affects thousands of individuals yearly, often manifesting in older adults in the seventh and eighth decades of life [1]. Treatment for CLL may include target drug therapy, chemotherapy, radiation, and surgery. Ibrutinib, a Burton’s tyrosine kinase (BTK) inhibitor, is FDA approved for the treatment of CLL as well as other conditions including mantle cell lymphoma and Waldenstrom's macroglobulinemia [2]. It works by binding irreversibly to BTK and inhibiting the proliferation of B cells and their survival [3]. Ibrutinib, therefore, takes advantage of the mechanism of action by which CLL proliferates. Ibrutinib has been identified to have largely mild adverse effects and has been a preferred treatment of choice, even in patients of poor physical status due to its general tolerability [4].

Common adverse effects of ibrutinib include diarrhea, increased risk of infections, lethargy, and cardiac manifestations, including an increased risk of atrial fibrillation [2]. Ibrutinib increases the risk of atrial fibrillation by up to 16% in patients undergoing therapy with this agent [5]. This is further complicated by the fact that ibrutinib has been found to interact with several antiarrhythmic agents which are used to treat atrial fibrillation. However, studies have found that approximately 85% of patients who develop atrial fibrillation are able to manage this condition and continue ibrutinib therapy [4-5]. It is theorized that by inhibiting BTK, ibrutinib prevents BTK from regulating the phosphoinositide 3-kinase and protein kinase B pathway which both have roles in protecting cardiac muscles [6]. Disruption of the protein kinase B pathway causes ibrutinib to be arrhythmogenic [6]. Bleeding is another complicating adverse effect of ibrutinib. Patients on ibrutinib therapy have a higher risk of bleeding due to platelet dysfunction secondary to inhibition of glycoprotein VI and glycoprotein 1b involved in platelet interaction with Von Willebrand factor [6]. This complicates the treatment of atrial fibrillation in patients on ibrutinib and the risk of stroke must be carefully balanced with the risk of bleeding prior to the decision to begin anticoagulation in these patients.

While many studies have explored the cardiac adverse effects of ibrutinib, little has been written about the cutaneous effects of ibrutinib. A rash occurs in up to 27% of patients with CLL treated with ibrutinib, though it is generally a nonpalpable petechial rash drug-induced by platelet dysfunction or a palpable pruritic rash from inhibition of epidermal growth factor receptor (EGFR) [7]. These rashes are mild and have not required patients to discontinue the use of ibrutinib therapy. In our literature review, there have been a few identified cases of ibrutinib causing severe vasculitis resulting in drug discontinuation, but no reports of a lichenoid drug reaction [8]. In this case report, we present a novel finding of severe lichenoid drug reaction experienced by a patient on ibrutinib therapy for CLL which required drug discontinuation. While Steven-Johnson syndrome (SJS) and drug rash with eosinophilia and systemic symptoms (DRESS) were considered initially, the lichenoid drug reaction was an unexpected finding in the patient. Please note, this article was previously presented as a meeting abstract at the Virtual Annual Scientific Session of the North Carolina Chapter of the American College of Physicians on February 11, 2022.

## Case presentation

**Figure 1:**
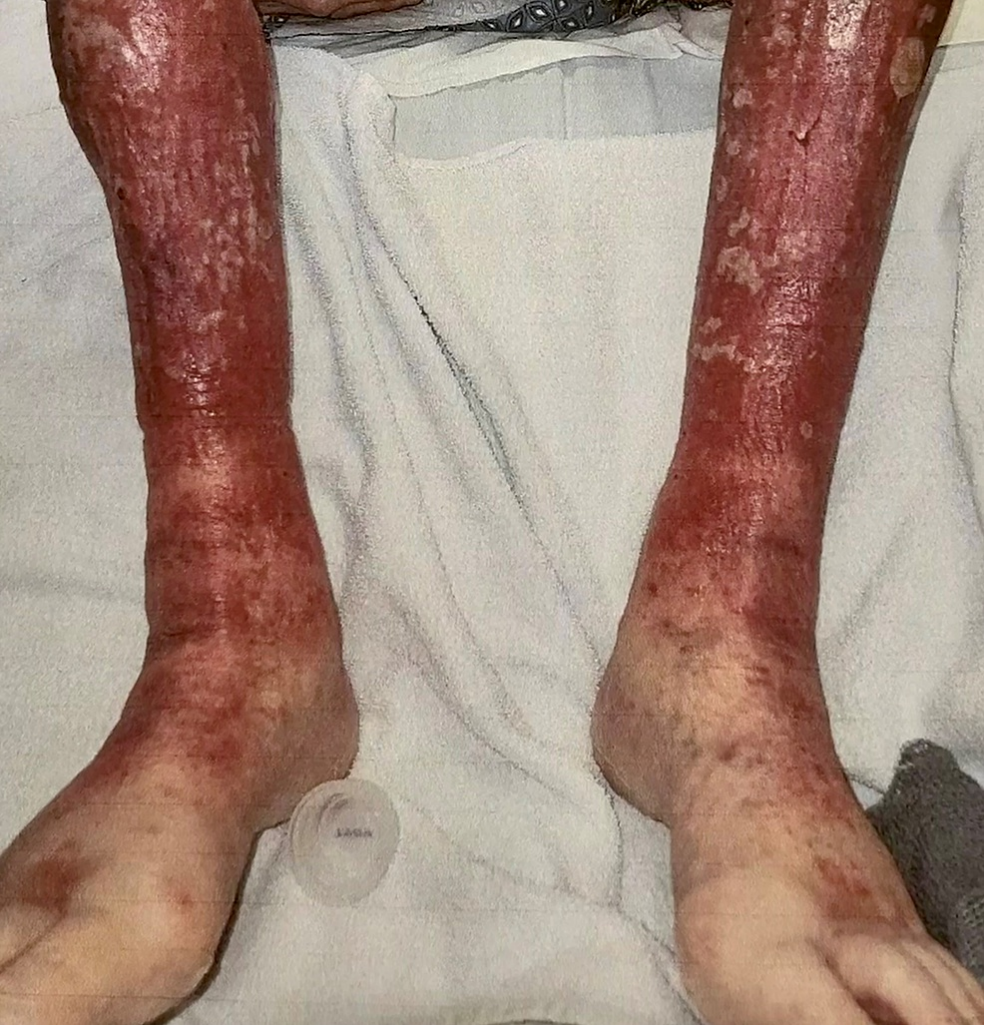
Patient’s rash on bilateral lower extremity

An 83-year-old male with a history of stage IV chronic lymphocytic leukemia, anemia, thrombocytopenia, and hyperlipidemia presented to the hospital with a blistering rash on his bilateral lower extremity. There was concern that ibrutinib may have caused this rash. His other home medications included rituximab, allopurinol, furosemide, fish oil, and a multivitamin. The patient had initiated ibrutinib therapy for CLL approximately 16 days prior to presentation to the hospital with this rash. His initial vitals on presentation were a temperature of 98.1 ⁰F, heart rate of 100, respiratory rate of 16, and oxygen saturation of 96% on room air. Physical exam showed a thin elderly male with mild conjunctival pallor and a rash on the bilateral lower extremities that was erythematous, raised, and with areas of vesicular eruption (Figure 1). Admission labs showed a white blood cell (WBC) count of 11.4 x 1000/mm3 with an absolute neutrophil count of 3.1 x 1000/mm3, absolute lymphocyte count of 8.1 x 1000/mm3, with no monocytes or eosinophils, hemoglobin of 10 g/dL, and a platelet count of 155 x 1000/mm3. A peripheral smear was obtained and showed mild leukocytosis with absolute lymphocytosis consistent with CLL. The basic metabolic panel (BMP) showed mild hyponatremia with a sodium of 131 mmol/L with the remainder of his basic metabolic panel (BMP) being within normal limits.

**Figure 2:**
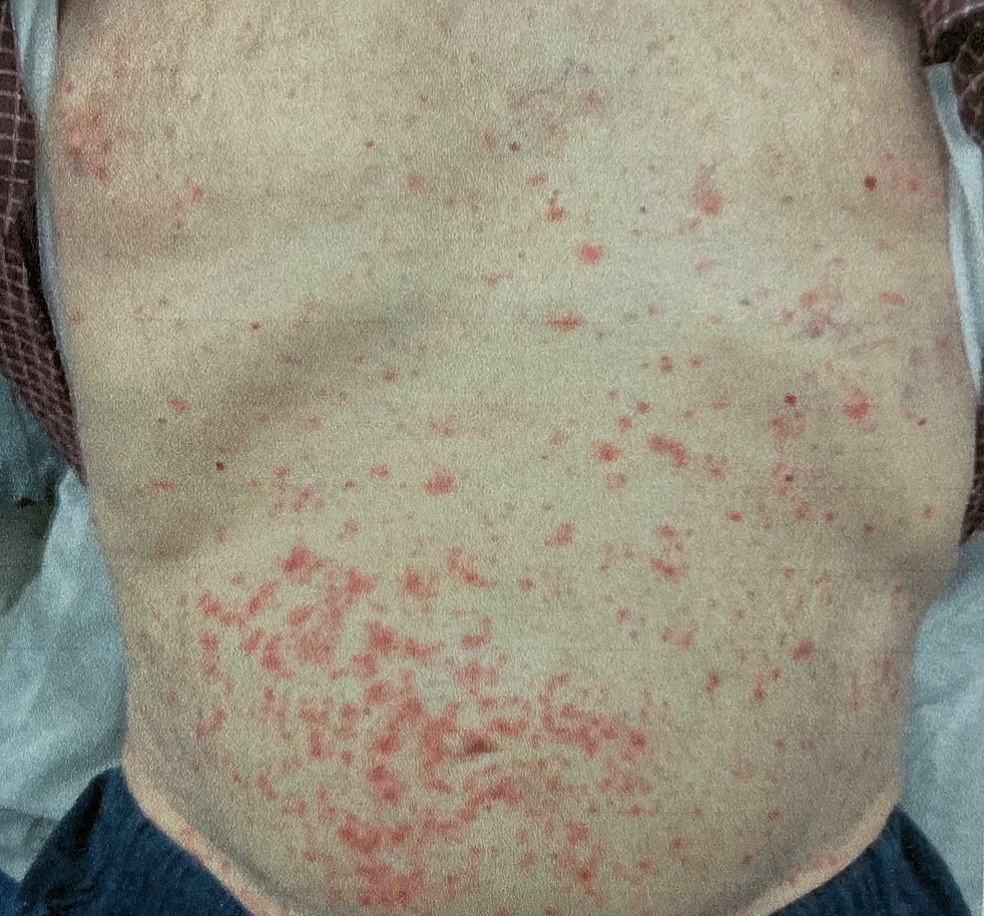
Patient’s rash on abdomen

The patient was admitted for management of his rash, with a concern that this may be SJS. Initially, the rash was confined to his bilateral lower extremities, but it quickly progressed to a maculopapular rash on his abdomen and back a few hours after admission (Figure 2). He did not have any mucosal involvement. The differential diagnosis for the rash included SJS, toxic epidermal necrolysis (TEN), DRESS, and vasculitis. The lack of mucosal involvement though was not consistent with SJS. Dermatology was electronically consulted, as no dermatology department was available physically on-site at that time.

Dermatology was highly concerned with the quick progression of the patient’s rash. Initially, the patient was started on 125 mg intravenous (IV) methylprednisolone and his legs were wrapped in ace wraps. He was fluid resuscitated with lactate ringers and a 4 mm punch biopsy of the patient’s skin from his abdomen was obtained. The patient then received IV methylprednisolone 60 mg daily, after the initial 125 mg dose. Urine eosinophils were also obtained and were negative. On day two of admission, the punch biopsy pathology showed a lichenoid drug reaction. 

**Figure 3:**
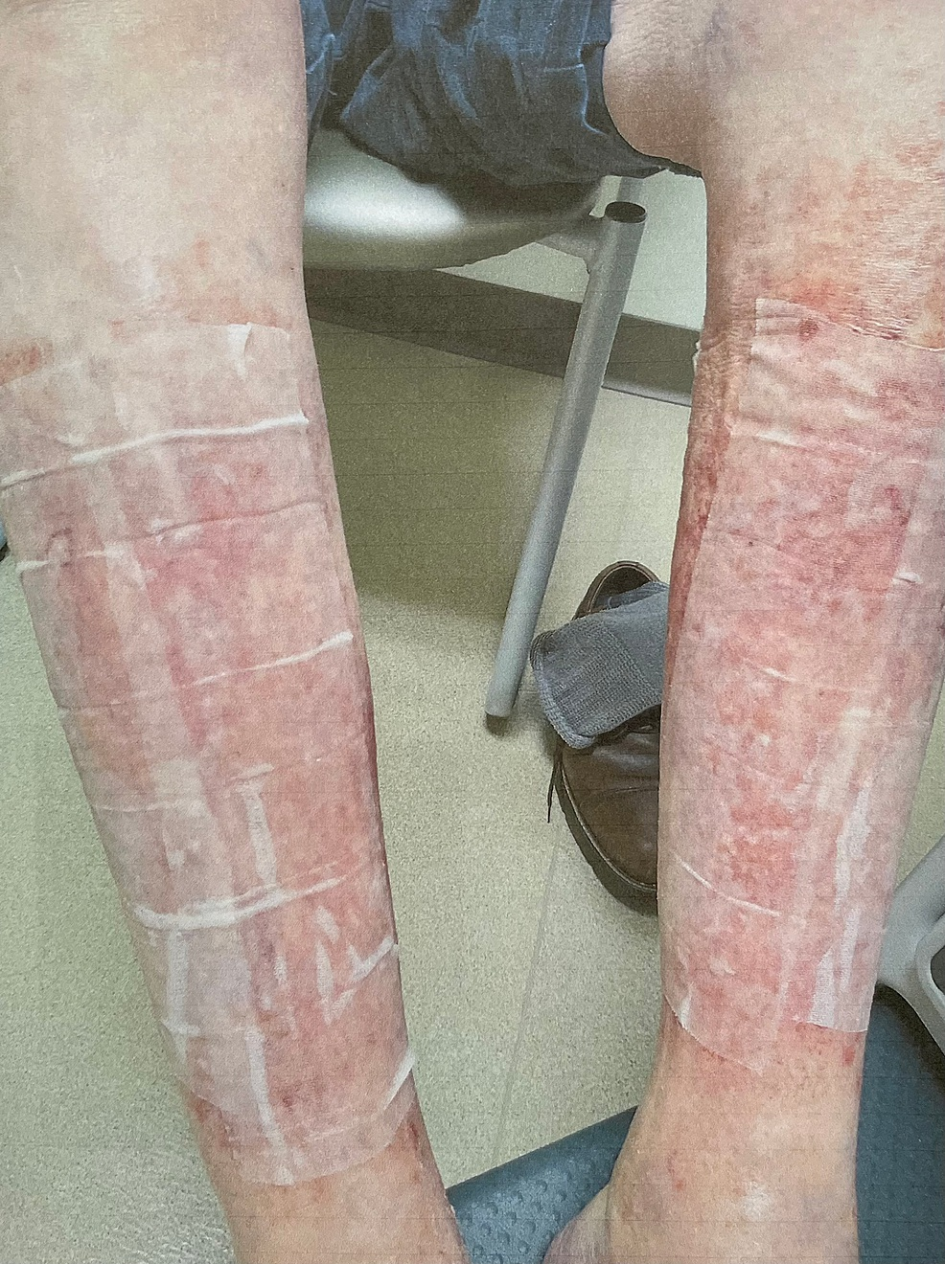
Patient’s rash appears improved at dermatology follow-up

The patient’s condition improved on the IV steroids, and he was discharged on day two of admission on an oral prednisone taper and topical triamcinolone cream with a follow-up appointment with dermatology. Upon follow-up with dermatology five days from discharge, the rash was noted to have significantly improved (Figure 3). At that visit, the patient was asked to stop using the triamcinolone cream but to continue the prednisone taper. He followed up with dermatology a month later and his rash had completely resolved at that time. The patient also followed up with his oncologist after discharge from the hospital. Due to the severity of the rash the patient had developed, the oncologist discontinued the ibrutinib therapy.

## Discussion

Chronic lymphocytic leukemia is a difficult disease to cure. The development of target drug therapies such as ibrutinib has significantly improved the treatment options for patients but a complete cure often remains elusive. Due to this, medications such as ibrutinib often become chronic therapies for patients suffering from CLL. Therefore, understanding the adverse effects of ibrutinib and the management of these effects becomes vital to ensure patients can tolerate this therapy.

A study examining the adverse effects and tolerability of ibrutinib noted that in over half of the patients who discontinue the drug over a four years period, the cause for discontinuation was drug toxicity [7]. While ibrutinib is a generally well-tolerated and highly efficacious drug of choice in the treatment of CLL, its adverse effects and toxicity can cause drug discontinuation after years of therapy.

The case presented in this article demonstrates a novel severe cutaneous drug reaction not previously recorded, to our knowledge. The lichenoid drug reaction experienced by this patient required discontinuation of therapy. Lichenoid drug reactions are uncommon reactions to medications characterized by erythematous papules and plaques, often found on extensor surfaces. The rash can clinically and histologically mirror the findings of lichen planus, but lichenoid drug reactions often have more perivascular involvement [9]. Management of lichenoid drug reactions includes drug discontinuation, topical corticosteroids, and oral or intravenous corticosteroid therapy if the reaction is severe [9].

Two important differential diagnoses in any patient presenting as the patient in this case did are SJS and TEN. SJS and TEN are rare and lethal drug reactions that result in blistering and necrosis of the skin, with SJS affecting less than 10% of the body surface and TEN greater than 30%. These conditions, which exist on a spectrum, are rapidly-progressive and should be on the differential diagnoses in any patient with severe cutaneous findings secondary to medication use. The treatment for SJS and TEN can involve supportive care, corticosteroids, intravenous immunoglobulin, and cyclosporine [10].

The development of target drug therapies in oncology has led to a new and improved way of treating cancers. However, each of these drugs has its own set of side effects. Crizotinib, an anaplastic lymphoma kinase (ALK) inhibitor, has been found to cause oral lichenoid lesions, while several other agents including erlotinib and imatinib have caused aphthous like ulcers [11]. However, beyond a rash caused by platelet dysfunction and a rash caused by inhibition of EGFR, ibrutinib has never been found to cause a lichenoid drug reaction in patients. Therefore, this case report highlights an important diagnosis to consider in patients receiving ibrutinib therapy and experiencing severe cutaneous adverse effects. Second-generation Burton’s tyrosine kinase inhibitors are being developed, with acalabrutinib and zanubrutinib currently available for use, which may offer improved side effect profiles compared to ibrutinib [12-15].

## Conclusions

Chronic lymphocytic leukemia is a disease affecting thousands of new patients yearly. Target drug therapies such as ibrutinib offer a chance for disease control in these patients. However, these target drug therapies are not without adverse effects. As patients initiate ibrutinib therapy, providers should have lichenoid drug reactions on the differential for any patient presenting with a blistering skin rash. Management of this rash will involve drug discontinuation and IV corticosteroid therapy as well as local skincare. There is certainly more to discover about the rare side effects that may come from target drug therapies in oncology. These adverse effects will need to be balanced by the benefits these medications may bring to patients as patients and care teams collaborate on treatment options.
